# Supplementary material for: Receptor-targeted engineered probiotics mitigate lethal Listeria infection
Source: Nat Commun. 2020 Dec 11;11:6344. doi: 10.1038/s41467-020-20200-5 (PMC7732855; doi:10.1038/s41467-020-20200-5)
Supplement: Supplementary file 1 — Supplementary Information [file 41467_2020_20200_MOESM1_ESM.pdf]

SUPPLEMENTARY INFORMATION

Receptor-targeted engineered probiotics mitigate lethal *Listeria* infection

Rishi Drolia[1,2], **Mary Anne Roshni Amalaradjou**[1,3], **Valerie Ryan**[1], **Shivendra Tenguria**[1,2], **Dongqi Liu**[1,2], **Xingjian Bai**[1], **Luping Xu**[1], **Atul K. Singh**[1], **Abigail D. Cox**[4], **Victor Bernal-Crespo**[4], **James A. Schaber**[5], **Bruce M. Applegate**[1,6], **Ramesh Vemulapalli**[4,7], and **Arun K. Bhunia**[1,2,4,6]*

[1]Molecular Food Microbiology Laboratory, Department of Food Science, Purdue University, West Lafayette, IN, USA

[2]Purdue Institute of Inflammation, Immunology and Infectious Disease, Purdue University, West Lafayette, IN, USA

[3]Department of Animal Science, University of Connecticut, Storrs, CT, USA

[4]Department of Comparative Pathobiology, Purdue University, West Lafayette, IN, USA

[5]Bindley Bioscience Research Center, Purdue University, West Lafayette, IN, USA

[6]Purdue University Interdisciplinary Life Science Program, Purdue University, West Lafayette, IN, USA

[7]Department of Veterinary Pathobiology, Texas A&M University, College Station, TX, USA

*bhunia@purdue.edu

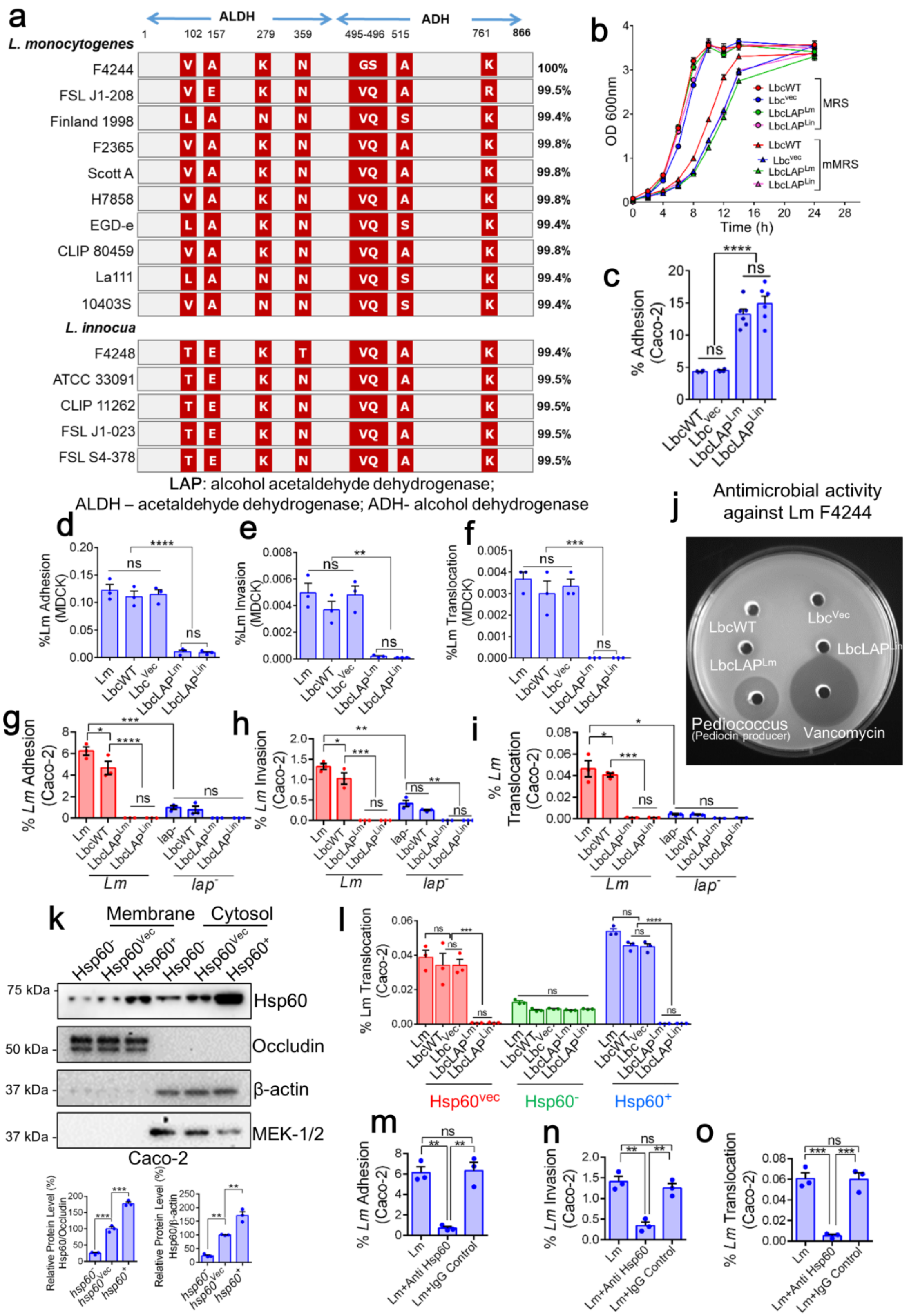

Supplementary Fig. 1 Analysis of LAP sequence, characterization of BLP-strains and Hsp60 expression in Caco-2 cells.

(a) Comparison of the amino acid sequence of LAP from different strains of *Lm* and *Lin* obtained from the NCBI database.

(b) Similar growth profiles of BLP strains in MRS and mMRS broths, relative to LbcWT or Lbc^{Vec} strains. (*n* = 3)

(c) Increased adhesion (MOE 10, 24 h) of the BLP strains (*n* = 6), relative to LbcWT (*n* = 4) strains or Lbc^{Vec} (*n* = 4) to Caco-2 cells.

(**d-f**, *n* = 3) Increased inhibition of *Lm* adhesion (**d**), invasion (**e**) and translocation (**f**) by the BLP strains in MDCK cells pre-exposed to *L. casei* strains for 24 h (MOE 10). *Lm* (MOI 50) was exposed for 1 h for adhesion and 2 h for invasion and translocation assays.

(**g-i**, *n* = 3) Increased inhibition of *Lm* (wild type) adhesion (**g**), invasion (**h**) and translocation (**i**) but not of the lap^{-}strain by the BLP strains in Caco-2 cells pre-exposed to *L. casei* strains for 24 h (MOE 10). *Lm* exposure as in **d-f**.

(j) Agar well diffusion assay showing the absence of bacteriocin-like antimicrobial activity in LbcWT, Lbc^{Vec} and BLP strains against *Lm* lawn. *Pediococcus acidilactici*, (pediocin; a bacteriocin producer) and vancomycin were used as positive controls.

(k) Immunoblot confirming Hsp60-knockdown ($Hsp60^{-}$) and- overexpression ($Hsp60^{+}$), relative to $Hsp60^{Vec}$ in Caco-2 cells. Occludin; membrane marker, and MEK-1/2; cytosolic marker. Bottom panels show normalized densitometry reports (*n* = 3).

(**l**) Increased inhibition of *Lm* translocation (*n* = 3) by the BLP strains in $Hsp60^{Vec}$ and $Hsp60^{+}$ but not in $Hsp60^{-}$ Caco-2 cells. *L. casei* pre-exposed for 24 h (MOE 10) before exposure to *Lm* for 2 h (MOI 50).

(m-o, *n* = 3) Decreased *Lm* adhesion (**m**), invasion (**n**) and translocation (**o**) in Caco-2 cells incubated with anti-Hsp60 mAb (5 µg/ml, 1 h) before *Lm* exposure. *Lm* exposure as in **d-f**.

Data in **b, c-i** and **k-o** represent the mean ± SEM with *n* = biologically independent samples from three independent experiments. The one-way (**c-f**, **k**, **m-o**) or two-way (**g-i**, **l**) ANOVA test followed by Tukey's multiple comparisons was used. For all analysis: ****p < 0.0001; ***p < 0.001; *p < 0.05; ns, no significance. Source data are provided as a Source Data file.

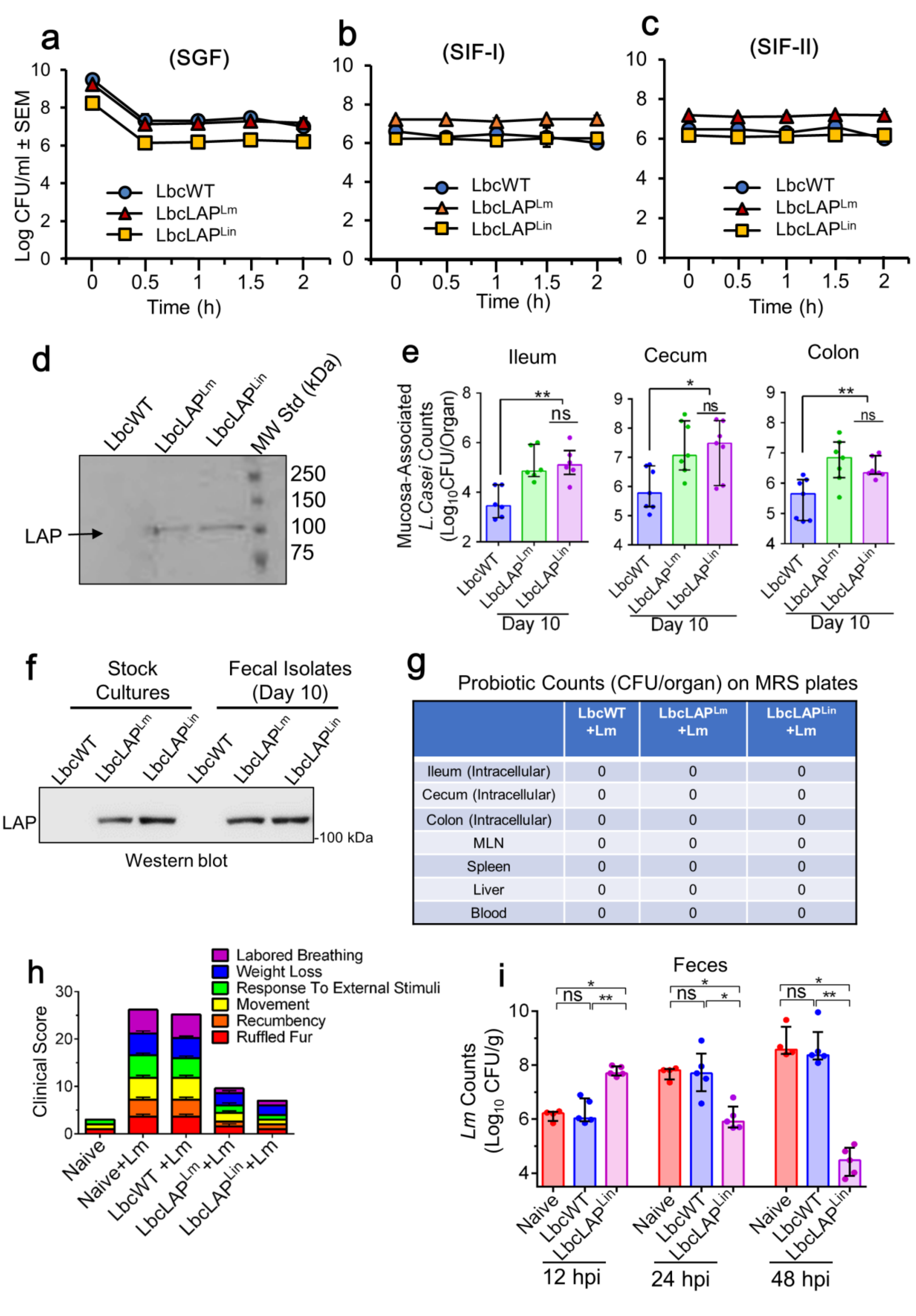

	LbcWT +Lm	LbcLAP^Lm +Lm	LbcLAP^Lin +Lm
Ileum (Intracellular)	0	0	0
Cecum (Intracellular)	0	0	0
Colon (Intracellular)	0	0	0
MLN	0	0	0
Spleen	0	0	0
Liver	0	0	0
Blood	0	0	0

Supplementary Fig. 2 ***In vitro*** **and** ***in vivo*** **characterization of BLP strains.**

(**a**-**c**) Similar survival of *L. casei* (LbcWT) and BLP strains in simulated gastric fluid (SGF) (**a**), simulated intestinal fluid I (SGF-I) (**b**), and simulated intestinal fluid II (SGF-II) (**c**). Data represent the mean of one single experiment with similar results from the three independent experiments.

(**d**) Immunoblot confirmation of LAP expression in BLP strains following sequential exposure to SGF, SIF-I, and SGF-II.

(**e**) Increased mucosal association of BLP strains in the ileum (*n* = 6), cecum (*n* = 7), and colon (*n* = 7), of mice on day 10. No colonies were detected on MRS+vancomycin plates from mock-treated control (naïve) animals. Each point represents an individual mouse (*n*) from three independent experiments.

(**f**) Immunoblot confirmation of LAP expression in fecal isolates of BLP strains.

(**g**) No BLP strains (10 days treatment) were detected in the intestinal (after gentamicin protection assay) or extra-intestinal tissue samples. Data are representative of *n = 7* mice from three independent experiments.

(**h**) Clinical score of mice after *Lm* challenge. Data represent mean ± SEM from *n = 5* representative mice from each treatment group of three independent experiments.

(i) Increased *Lm* shedding in the feces in mice treated with BLP-strains at 12 hpi (*n* = 4, 5, 5, for each group, respectively) but significantly reduced at 24 (*n* = 4, 5, 5, for each group, respectively) and 48 (*n* = 4, 5, 5, for each group, respectively) hpi. Each point represents an individual mouse (*n*) from two independent experiments.

Bar and brackets in **e** and **i** represent the median and interquartile range, respectively. The Mann-Whitney nonparametric test (two-tailed) was used, and comparisons were made between each treatment group individually. For all analysis: $^{**}p < 0.01$; $^{*}p < 0.05$; ns, no significance. Panels **d** and **f** are representative of three independent experiments. Source data are provided as a Source Data file.

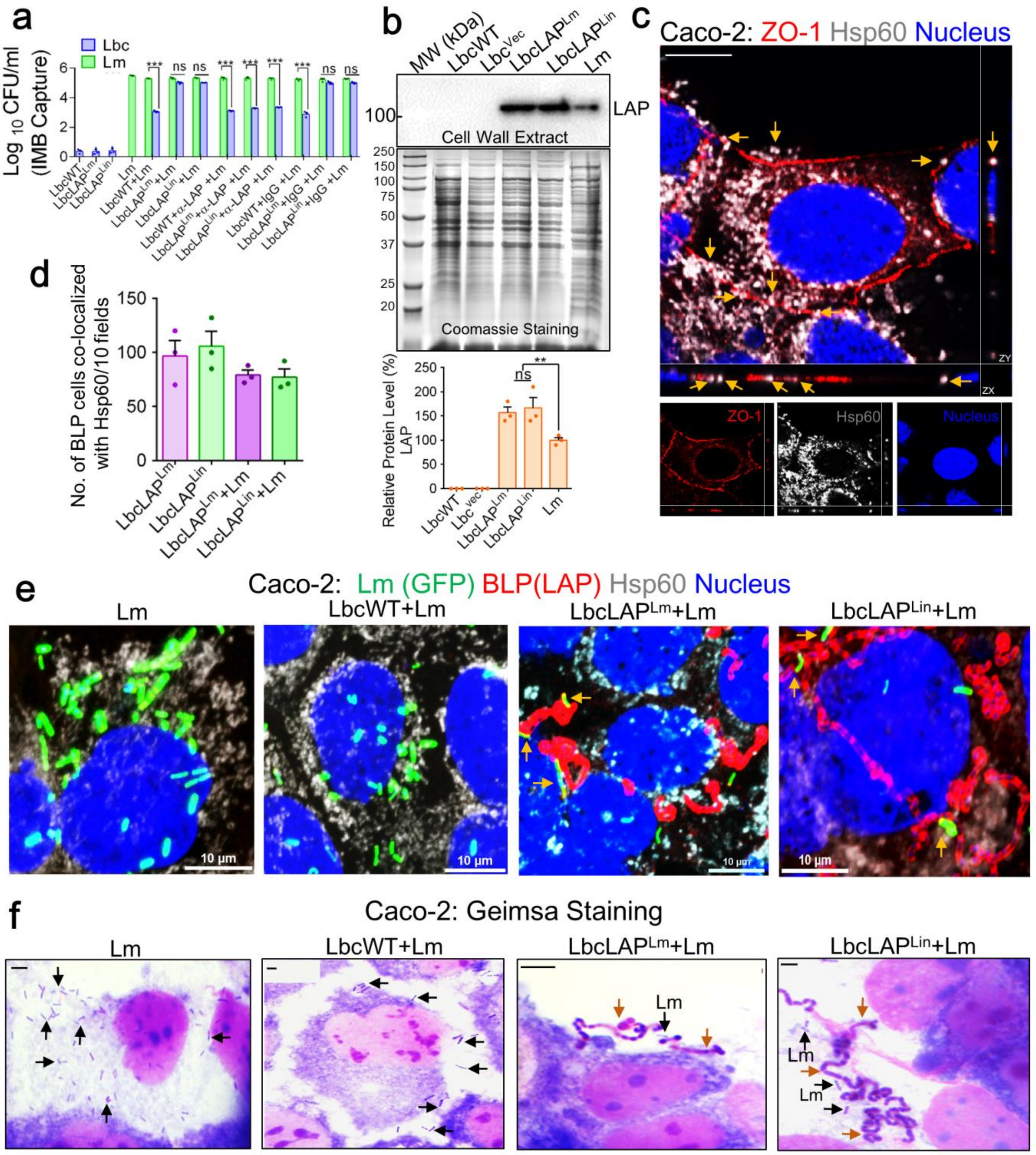

Supplementary Fig. 3 Co-aggregation and surface co-localization of BLP with Hsp60.

(**a**) Inhibition of formation of BLP-*Lm* co-aggregates (CFU, mean ± SEM, *n* = 3 from three independent experiments) by pre-incubation of BLP with anti-LAP mAb but not with an isotype IgG followed by IMB-based capture.

(**b**) Western blot showing relative expression of LAP on BLP and *Lm* cell wall. Equal amounts of proteins are analyzed (BCA assay) as confirmed by Coomassie blue staining (bottom panel). The densitometry report (bottom-most panel) represent mean ± SEM, $n = 3$ from three independent experiments.

(**c**) Micrographs of Caco-2 cells immunostained for ZO-1 (red, cell periphery), Hsp60 (white, host cell receptor) and nucleus (blue; DAPI). Surface expression of Hsp60 (white) and co-localization with ZO-1 (red) were evident on the Caco-2 cell membrane (puncta, yellow arrows). Separated channels; bottom panels. Bars, 10 µm.

(**d**) Measurements (mean ± SEM) of co-localized BLP cells with membrane expressed Hsp60 from immunostained images (**Fig. 4 e**-**h**). Each point represents an average of 10 fields from each of the three independent experiments n = 30 fields.

(**e**) Micrographs of *Lm*-GFP cells co-incubated with or without *L. casei* (LbcWT and BLP) strains on Caco-2 cells (1:1 ratio, MOE 50 for each, 1 h). Cells were immunostained for LAP on BLP strains (red), Hsp60 (white, host cell receptor) DAPI (blue, nucleus) and *Lm*-GFP cells (green). Bars, 10 µm. In BLP co-incubated cells the *Lm*–GFP cells showed markedly reduced adhesion and BLP cells were co-aggregated with *Lm*–GFP cells (arrows). No immunostaining of LbcWT was observed with the anti-LAP mAb.

(**f**) Giemsa staining micrographs depicting reduced adhesion and competitive exclusion of *Lm* (black arrow) cells by BLP strains (yellow arrows) but not by LbcWT strains. *Lm* cells with or without *L. casei* (LbcWT and BLP strains) strains were co-incubated (1:1 ratio, MOE 50 for each, 1 h) on Caco-2 cells. Bars, 1 µm.

For plots, **a, b** and **d**, the one-way ANOVA test followed by Tukey's multiple comparisons was used. For all analysis: ***$p < 0.001$; **$p < 0.01$; ns, no significance. All images in **c, e, f** are representative of five different fields from three independent experiments. Source data are provided as a Source Data file.

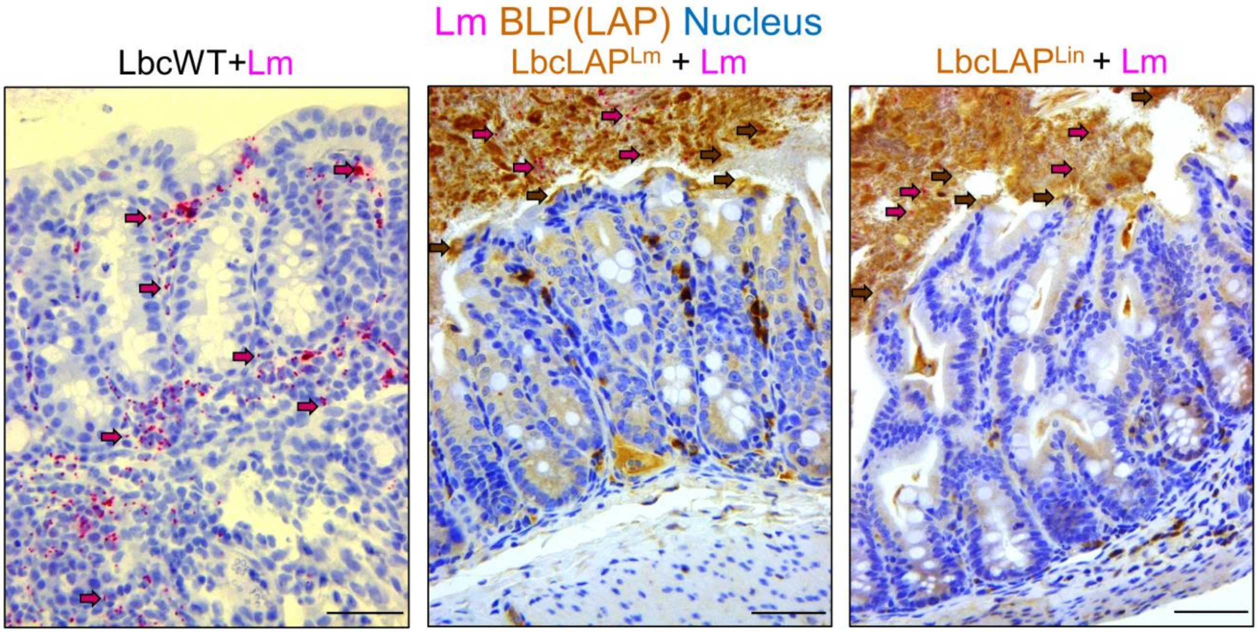

Supplementary Fig. 4 BLP and *Lm* co-aggregation on colonic villi.

Zoomed-out micrographs of colonic villi of LbcWT-or BLP-treated (10 days) mice at 48 hpi dual immunostained for *Listeria* (anti-*Lm* pAb, pink rods, pink arrows) and LAP (anti-LAP mAb to stain the BLP strains, brown, brown arrows) and counterstained with hematoxylin to stain the nucleus (blue). Bars, 25 µm. Translocated *Lm* is observed in the lamina propria (pink arrows, left panel) in LbcWT-treated mice but confined in the lumen (pink arrows, middle and right panels) in BLP-treated mice. Images are representative of 10 different fields from 4 independent mice for each treatment. Source data are provided as a Source Data file.

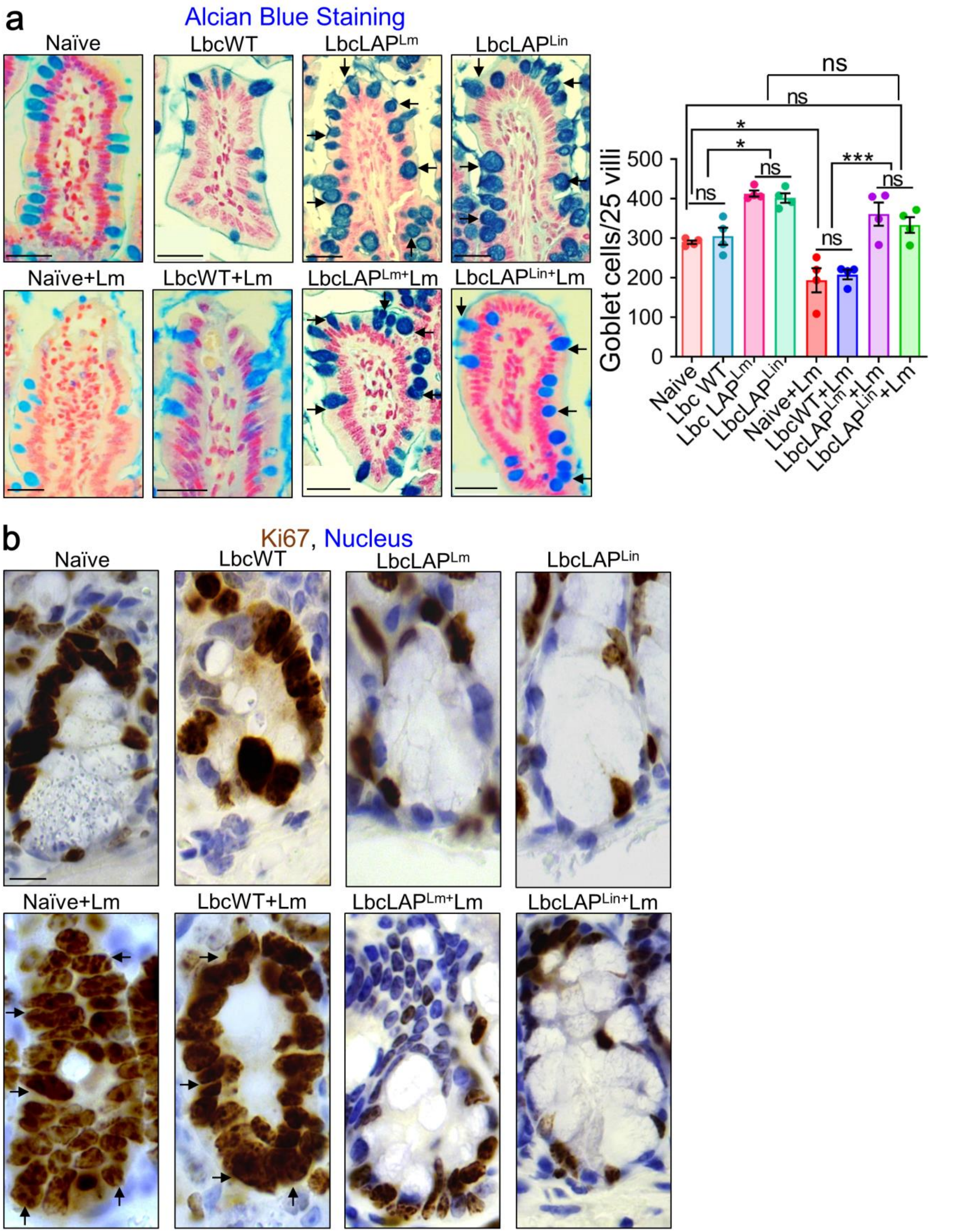

Supplementary Fig. 5 Goblet cell counts and crypt cell proliferation in ileal villi.

(**a**) Representative Alcian blue staining micrographs of ileal tissue sections from control (mock-treated) uninfected naïve mice, *L. casei*-treated (10 days, LbcWT or BLP) pre or post-*Lm* challenge at 48 hpi depicting increased goblet cells (arrows) in BLP-treated mice and quantification of goblet cells (right panel, each point represents an individual mouse, 4 mice per group, *n* = 100 villi). Scale bar, 25 µm. Data represent the mean ± SEM and statistical significance was determined by using the one-way ANOVA test followed by Tukey's multiple comparisons. For analysis: ***$p < 0.001$; *$p < 0.05$; ns, no significance. Source data are provided as a Source Data file.

(**b**) Representative immunohistochemical micrographs (high magnification) of the ileal crypt stained for Ki67 (brown) from control (mock-treated) uninfected naïve mice or *L. casei*-treated (10 days, LbcWT or BLP) pre-or post-*Lm* challenge at 48 hpi. Arrows depict increased Ki67^{+} cells in the naïve of LbcWT-treated mice at 48 hpi. Scale bar, 10 µm. Images (a, b) are representative of four independent mice.

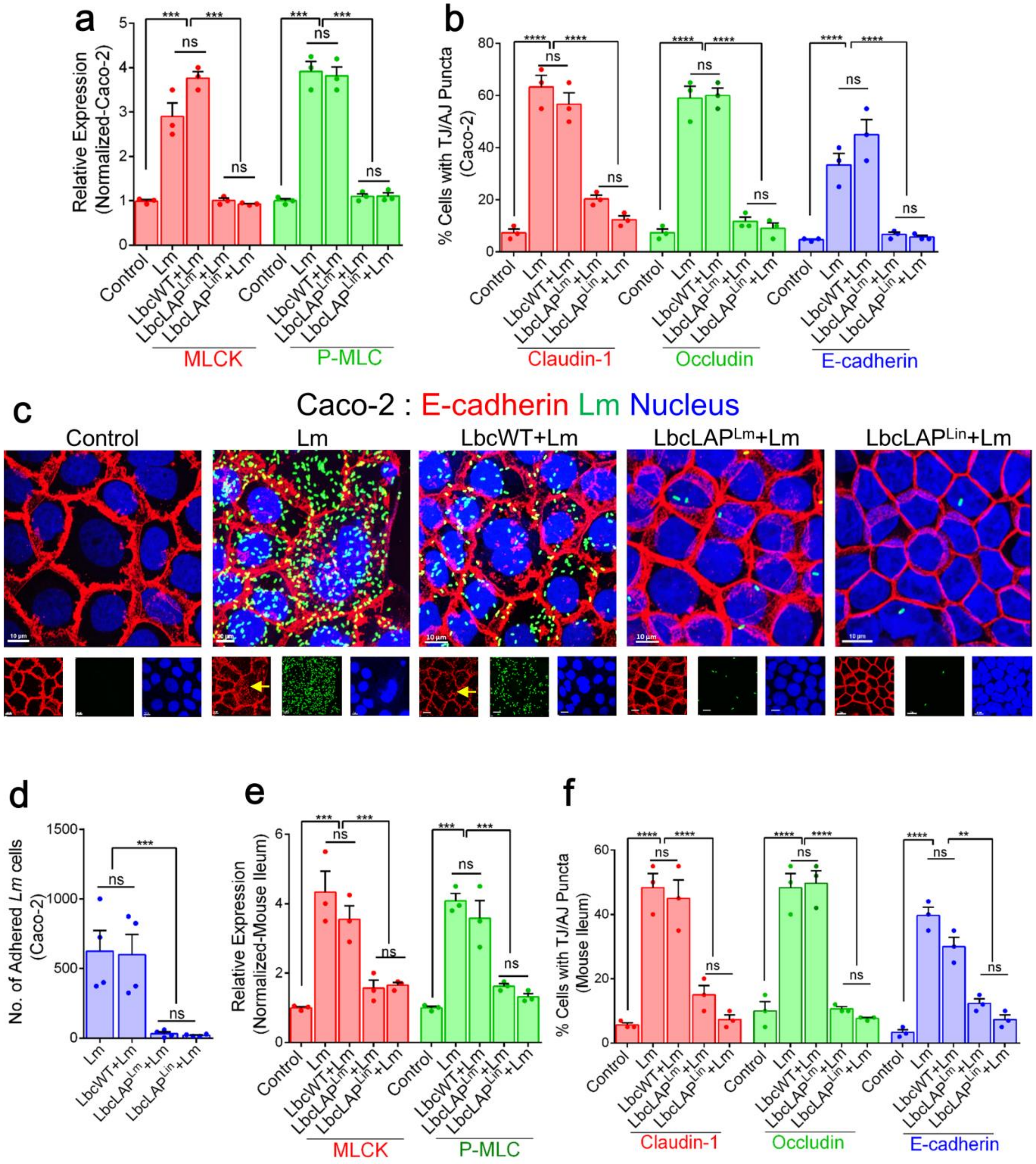

Supplementary Fig. 6 BLP preserves *Lm*-induced disturbance of intestinal epithelial cell-cell junctional integrity.

(**a**, **b**) Quantitative analysis (mean ± SEM, *n* = 3) of MLCK and P-MLC expression (**a**) and of claudin-1, occludin, and E-cadherin puncta formation (**b**) from images of immunostained Caco-2 cells (**Fig. 7e**) treated with LbcWT or BLP strains (MOE; 10, 24 h) before exposure with *Lm* (MOI; 50, 2h).

(**c**) Separated channel images showing mislocalization (intracellular puncta, endocytosis) of E-cadherin (labeled in red; arrows) in *Lm* exposed cells or treated with LbcWT before *Lm* exposure (MOI; 50, 2h). Caco-2 cells pretreated with BLP strains show intact localization of E-cadherin and markedly reduced adhesion of *Lm*. Separated channels are shown individually at the bottom of the merged images for clarity of the E-cadherin panel in **Fig. 7e**. Images are representative of five different fields from three independent experiments.

(**d**) Enumeration (mean ± SEM, *n* = 4 independent biological samples) of adhered *Lm* cells from immunostained images (**Fig. 7e**) of Caco-2 cells treated with LbcWT or BLP strains (MOE 10, 24 h) before *Lm* exposure (MOI 50, 2h).

(**e**, **f**) Quantitative analysis (mean ± SEM, *n* = 3 mice) of MLCK and P-MLC expression (**e**) and of claudin-1, occludin, and E-cadherin puncta formation (**f**) from images of immunostained ileal tissues (**Fig. 7f**) of mock-treated uninfected naïve mice or naïve, LbcWT or BLP-treated mice (10 days) at 48 hpi with *Lm*.

Each point represents an average of five different fields from one of the three (**a, b)** or four (**d**) independent experiments or a single mouse per treatment (**e, f**) The one-way (**d**) or two-way ANOVA (**a, b, e** and **f**) test followed by a Tukey's multiple comparisons was used. For all analysis: ****p < 0.0001; ***p < 0.001; **p < 0.01; ns, no significance. Source data are provided as a Source Data file.

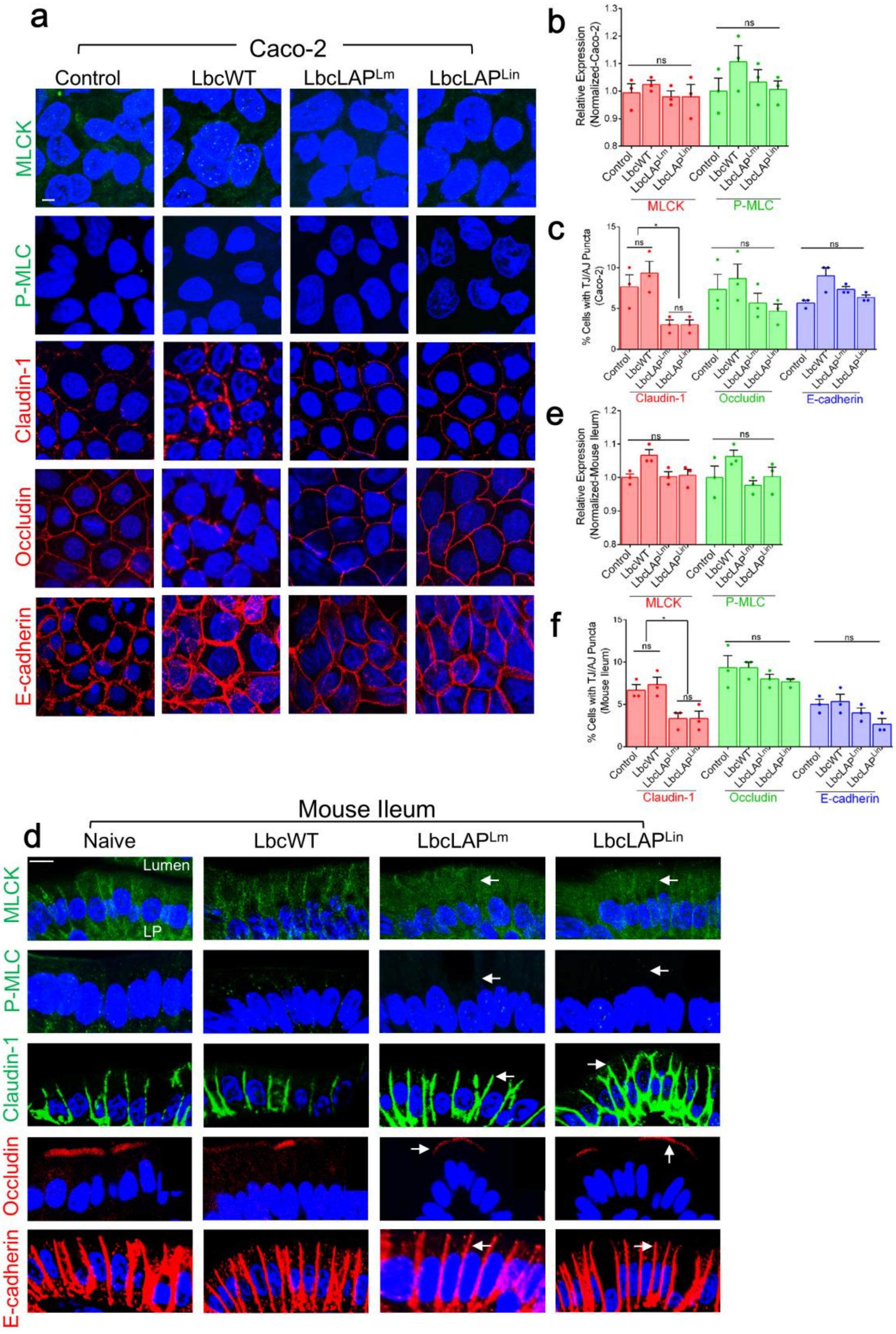

Supplementary Fig. 7 BLP does not cause disturbance of intestinal epithelial cell-cell junctional integrity.

(**a**, **d**) Confocal immunofluorescence micrographs of immunostained Caco-2 cells without treatment (control) or exposed to LbcWT or BLP strains (MOE 10, 24 h) without *Lm* exposure (**a**) or immunostained ilea from mock-treated mice (naïve) or treated with LbcWT or BLP strains (10 days) without *Lm* exposure (**d**) showing baseline MLCK (green, arrows, **d**) and P-MLC (green, arrows, **d**) expression and intact localization of claudin-1 (red) in Caco-2 (A), green in mouse ileum (**d**, arrows), occludin (red, arrows, **d**), and E-cadherin (red, arrows, **d**). Images are representative of five different fields from three independent experiments (**a)** or 3 mice per treatment (**d**). Scale bars, 10 µm.

(**b**, **c**, **e**, **f**) Quantitative analysis (mean ± SEM, n = 3 biologically independent samples) of MLCK and P-MLC expression in Caco-2 (**b**) and mouse ileum (**e**) and of claudin-1, occludin, and E-cadherin puncta formation in Caco-2 (**c**) and mouse ileum (**f**) from images of immunostained Caco-2 cells (**a**) or mouse ileum (**b**). Each point represents an average of five different fields from one of the three independent experiments (**b, c**) or a single mouse per treatment (**e, f**).

Statistical significance was determined by using the two-way ANOVA (**b, c, e** and **f**) test followed by Tukey's multiple comparisons. $*p < 0.05$; ns, no significance. Source data are provided as a Source Data file.

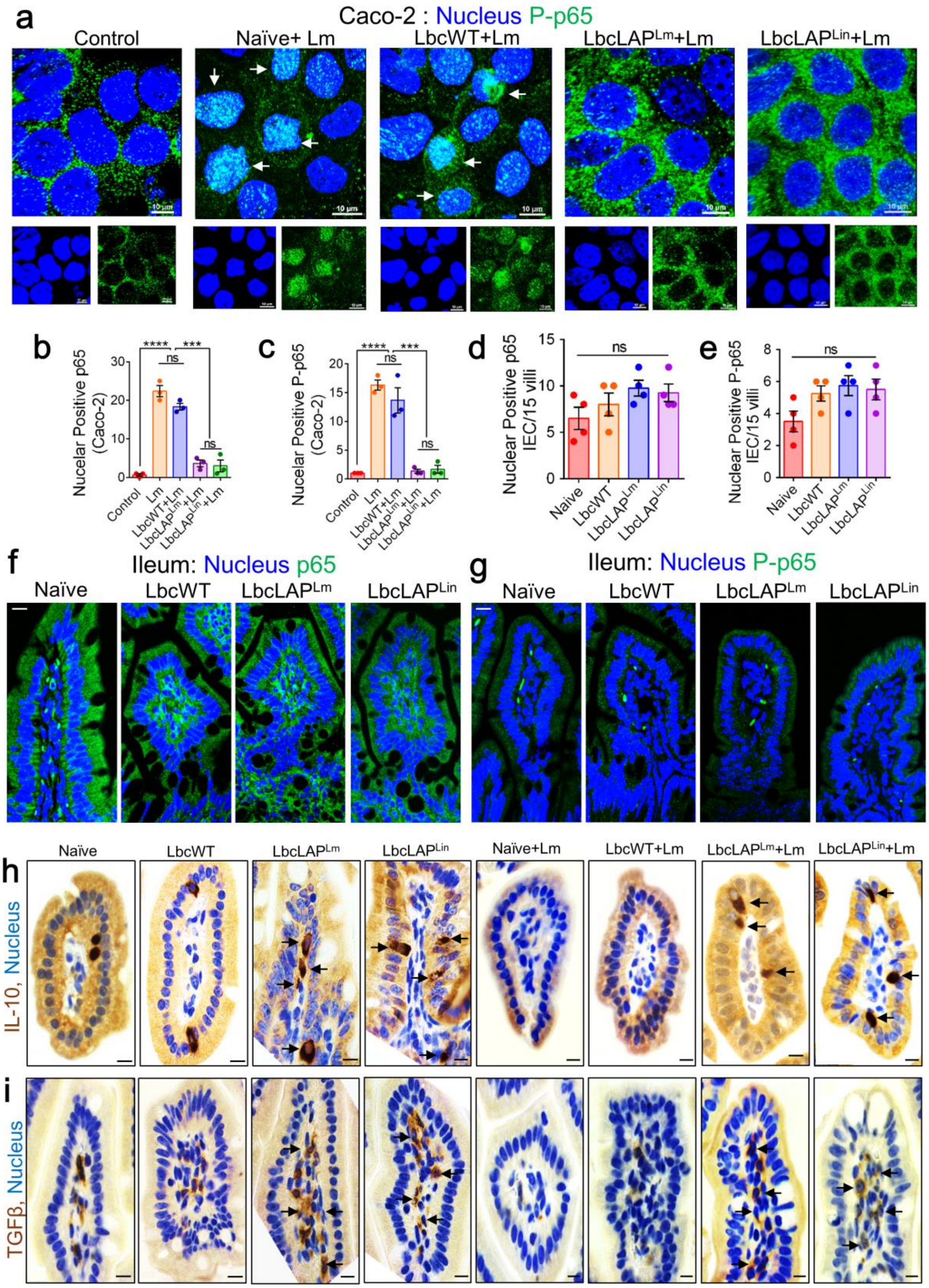

Supplementary Fig. 8 BLP prevents *Lm*-induced NF-κB activation and modulates cytokines.

(**a**) Confocal immunofluorescence micrographs showing decreased nuclear localization of P-p65 (**a**, green) in Caco-2 cells treated with BLP strains (MOE 10, 24 h) but not with LbcWT before *Lm* exposure (MOI 50, 1h). Arrows indicate the nuclear localization of P-p65 in *Lm* exposed cells or those treated with LbcWT strain before *Lm* exposure. Separated channels are shown individually in the bottom panels of the merged images. Scale bars, 10 µm. Images are representative of five different fields from three independent experiments.

(b, **c**) Quantified results from **Fig. 8a** expressed as mean ± SEM p65 and **Supplementary Fig. 8b** expressed as mean ± SEM P-p65 (**b**) nuclear positive Caco-2 cells. Each point represents five different fields from one of the three independent experiments (*n* = 15 fields).

(**d**, **e**, **f**, **g**) Quantified results (mean ± SEM) and confocal immunofluorescence micrographs and showing no increase in nuclear localization of p65 (**d**, **f**) and P-p65 (**e**, **g**) in villi of mice ilea after *L. casei* treatment (LbcWT or BLP) for 10 days, relative to naïve mock-treated mice. Each point in **d**, **e** represents an average of 15 villi from a single mouse, 4 mice per group, *n* = 60 villi. Scale bars, 10 µm.

(**h**, **i**) Representative immunohistochemical micrographs of sections of the ileum showing increased IL-10^+ cells (**a**, brown, arrows) and TGFβ$^+$ cells (**b**, brown, arrows) in BLP-treated (10 days) mice, relative to LbcWT-treated (10 days) mice pre or post-*Lm* challenge at 48 hpi. Nuclei are counterstained (blue). Scale bars, 10 µm

Statistical significance (**b**, **c**, **d** and **e)** was determined by using the one-way ANOVA followed by Tukey’s multiple comparisons test. For all analysis: ****p < 0.0001; ***p < 0.001; ns, no significance. Source data are provided as a Source Data file.

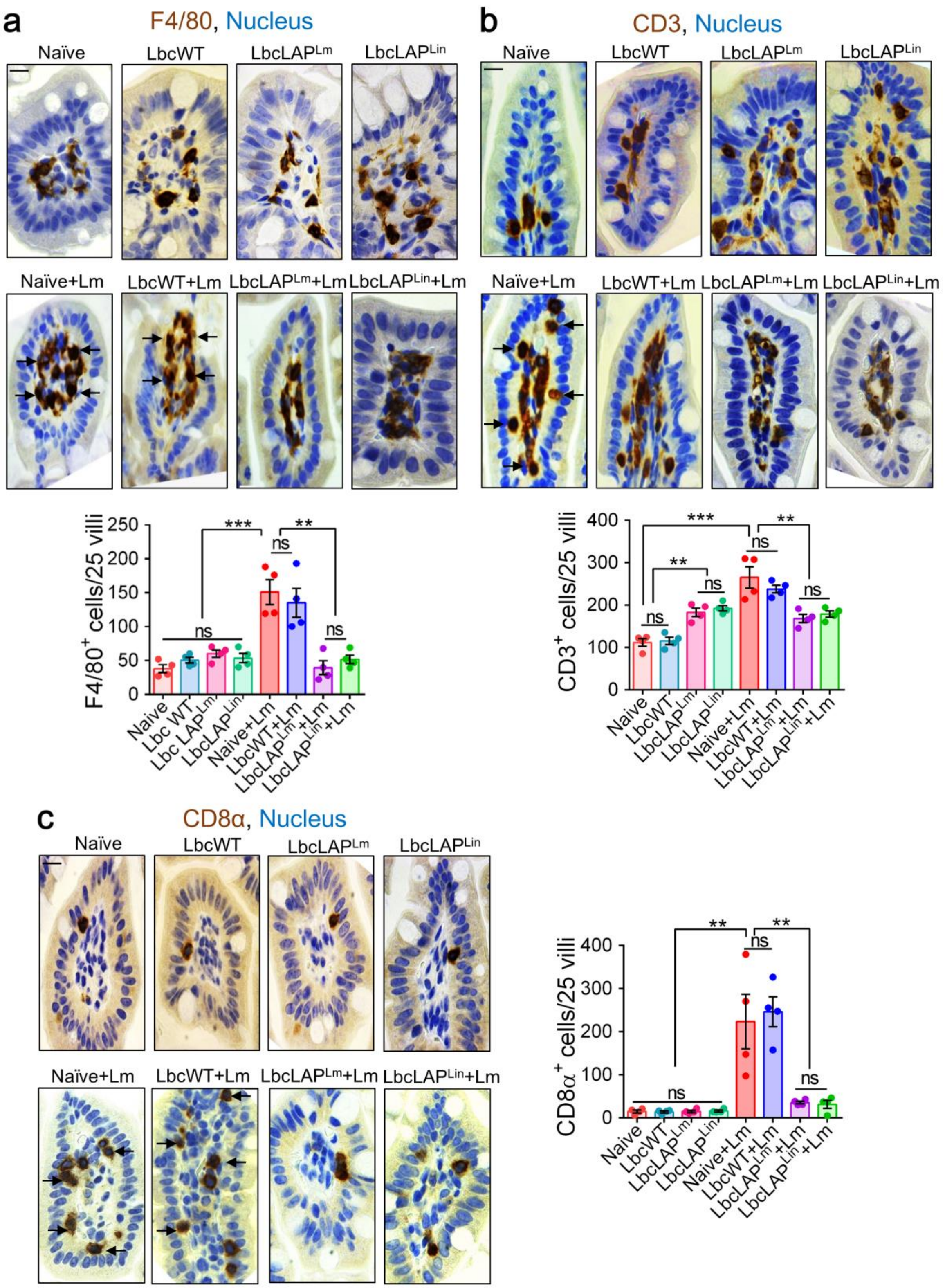

Supplementary Fig. 9 Analysis of F4/80 (macrophage), CD3 and CD8 positive cells in the intestinal tissues of BLP-treated mice.

(**a, b**) Representative immunohistochemical micrographs (top panels) and respective quantification (bottom panels, mean ± SEM) of ileal tissues showing increased F4/80$^+$ macrophage (**a**, brown, arrows), CD3$^+$ cells (**b,** brown, arrows) in naïve or LbcWT-treated (10 days), relative to BLP-treated (10 days) mice at 48 hpi. Bars, 10 µm.

(c) Representative immunohistochemical micrographs (left) and respective quantification (right, mean ± SEM) of ileal tissues showing increased CD8$^+$ cells (**a**, brown, arrows) in naïve or LbcWT-treated (10 days), relative to BLP-treated (10 days) mice at 48 hpi. Bars, 10 µm.

For panels **a-c** tissue sections were counterstained with hematoxylin to stain the nucleus. Each point represents an average of 25 villi from a single mouse, 4 mice per group, *n* = 100 villi. Statistical significance was determined by using the one-way ANOVA followed by Tukey's multiple comparisons test. For all analysis: ***p < 0.001; **p < 0.01; ns, no significance. Source data are provided as a Source Data file.

Supplementary Table 1. List of antibodies, bacterial strains, biological samples, chemicals, critical commercial assays, experimental models (cell lines and mouse strains), oligonucleotides and software/algorithms used in the study.

Reagents	Source	Catalog No.
**Antibodies**		
Mouse monoclonal anti-LAP	Our Lab[1]	N/A
Horse anti-mouse IgG (HRP-linked)	Cell Signaling	Cat # 7076, RRID: AB_330924
Goat anti-rabbit IgG (HRP-linked)	Cell Signaling	Cat # 7074, RRID: AB_2099233
Rat monoclonal anti-ZO-1	Thermo Fisher Scientific	Cat # MABT11MI, RRID: AB_628459
Rabbit polyclonal anti-*Listeria*	Our Lab	N/A
Rabbit polyclonal anti-InlA	Our Lab[2]	N/A
Mouse monoclonal anti-N-acetylmuramidase (NamA)	Our Lab[3]	N/A
Rabbit polyclonal anti-Hsp60	Our Lab[2]	N/A
Mouse monoclonal anti-β-actin	Santa Cruz Biotechnology	Cat # sc-47778, RRID: AB_626632
Normal mouse IgG	Santa Cruz Biotechnology	Cat # sc-2025, RRID: AB_737182
Normal rabbit IgG	Santa Cruz Biotechnology	Santa Cruz Biotechnology
Mouse monoclonal anti-Hsp60	Enzo life Science	Cat # ADI-SPA-806F, RRID: AB_11177888
Rabbit monoclonal anti-MEK 1/2	Cell Signaling	Cat # 8727, RRID:AB_10829473
Rabbit polyclonal anti-Muc-2	Novus Biological	Cat # NBP1-31231, RRID: AB_10003763
Rabbit monoclonal anti- Ki67	Cell Marque	Cat # 275R-15, RRID: AB_1158033
Rabbit polyclonal anti-Cleaved-caspase 3 (Asp175)	Cell Signaling	Cat # 9661S, RRID: AB_2341188
Mouse monoclonal anti-MLCK	Sigma-Aldrich	Cat # M7905, RRID: AB_477243
Rabbit polyclonal anti–phosphorylated MLC	Cell Signaling	Cat # 3671, RRID: AB_330248
Mouse monoclonal anti–phosphorylated MLC	Cell Signaling	Cat # 3675, RRID: AB_2250969
Mouse monoclonal anti-Claudin-1	Thermo Fisher Scientific	Cat # 37-490-0, RRID: AB_ 2533323
Mouse monoclonal anti-Occludin	Thermo Fisher Scientific	Cat # 33-150-0, RRID: AB_2533101
Rabbit monoclonal anti-E-cadherin (Human)	Cell Signaling	Cat # 3195, RRID: AB_2291471
Rat monoclonal anti-E-cadherin (Mouse)	Invitrogen	Cat # 13-190-0, RRID: AB_2533005

Rabbit monoclonal anti-NF-κB p65	Cell Signaling	Cat # 8242, RRID: AB_10859369
Rabbit monoclonal anti-Phospho-NF-κB p65 (Ser536)	Cell Signaling	Cat # 3033, RRID: AB_331284
Rat monoclonal anti-IL-10	Abcam	Cat # AB 189392, RRID: AB_733113
Rat monoclonal anti-TGF-β clone (EPR21143)	Abcam	Cat # AB215715, RRID: NA
Rat monoclonal anti-F4/80	Bio-Rad	Cat # MCA497R, RRID: AB_323279
Rat monoclonal anti-CD4	Invitrogen	Cat # 14-9766-82, RRID: AB_2573008
Rabbit polyclonal anti-CD3	Dako	Cat # A0452, RRID: AB_ 2335677
Rat monoclonal anti-CD8α	Invitrogen	Cat # 14-0808-82, RRID: AB_2572861
Rat monoclonal anti-FoxP3	Invitrogen	Cat # 14-5773-82, RRID: AB_467576
Rabbit polyclonal anti-CD11c	Invitrogen	Cat # PA5-79537, RRID: AB_ 2746652
Goat polyclonal anti-NKp46	R&D Systems	Cat # AF2225, RRID: AB_ 355192
Goat anti-mouse IgG (H+L), F(ab')2 Fragment (Alexa Fluor 488 Conjugate) antibody	Cell Signaling	Cat # 4408, RRID: AB_10694704
Goat anti-rabbit IgG (H+L), F(ab')2 Fragment (Alexa Fluor 488 Conjugate) antibody	Cell Signaling	Cat # 4412, RRID: AB_1904025
Goat anti-mouse IgG (H+L), F(ab')2 Fragment (Alexa Fluor 555 Conjugate) antibody	Cell Signaling	Cat # 4409, RRID: AB_1904022
Goat anti-rabbit IgG (H+L), F(ab')2 Fragment (Alexa Fluor 555 Conjugate) antibody	Cell Signaling	Cat # 4413, RRID: AB_10694110
Goat anti-rat IgG (H+L), (Alexa Fluor 555 Conjugate) antibody	Cell Signaling	Cat # 4417, RRID: AB_10696896
Goat anti-rabbit IgG (H+L), Highly Cross-Adsorbed (Alexa Fluor 647 Conjugate) antibody	Invitrogen	Cat # A21245, RRID: AB_253813
Goat anti-rabbit ImmPRESS HRP	Vector Labs	Cat # MP-7451, RRID: AB_ 2631198
Goat anti-rat ImmPRESS HRP (M adsorbed)	Vector Labs	Cat # MP-7444, RRID: AB_ 2336530
Horse anti-mouse ImmPRESS HRP	Vector Labs	Cat # MPX-2402, RRID: AB_ 2336831
Horse anti-Goat ImmPRESS HRP	Vector Labs	Cat # MP-7405, RRID: AB_ 2336526
Isotype control Mouse IgG	Vector Labs	Cat # I-2000, RRID: AB_ 2336354
Isotype control Rabbit IgG	Vector Labs	Cat # I-1000, RRID: AB_ 2336355

Isotype control Rat IgG	Vector Labs	Cat # I-4000, RRID: AB_ 2336356
**Bacterial Strains**		
L. monocytogenes F4244 (WT), serovar 4b, a clinical isolate	CDC, Atlanta, GA[3]	N/A
L. monocytogenes KB208 (*lap*⁻)	Our Lab[4]	N/A
L. monocytogenes CKB208 (lap^{-}+lap^{Lm})	Our Lab[4]	N/A
L. monocytogenes CKB208 (lap^{-}+lap^{Lin})	This study	N/A
L. monocytogenes F4244 expressing GFP	Bruce Applegate Lab	N/A
L. innocua F4248	CDC, Atlanta, GA	N/A
Lactobacillus casei, ATCC334 (WT)	Michael Miller, UIUC	N/A
Lactobacillus casei, vancomycin-resistant (300 µg/ml)	This study	N/A
L. casei expressing LAP of *Lm* (AKB906, $LbcLAP^{Lm}$)	This study	N/A
L. casei expressing LAP of *L. innocua* (AKB907, $LbcLAP^{Lin}$)	This study	N/A
L. casei carrying empty plasmid pLP401T (Lbc^{Vec})	This study	N/A
Pediococcus acidilactici strain H	Our Lab[5]	N/A
Salmonella enterica serovar Typhimurium ver. Copenhagen	Our Lab	N/A
**Biological Samples**		
Tissues isolated from ileum and colon of 8-10 weeks-old A/J mice	N/A	N/A
**Chemicals**		
MRS Broth	Difco/BD	Cat # D088107
Modified MRS containing 1% mannitol	Koo et al. 2012[6]	N/A
Modified Oxford agar Base	Neogen Corporation	Cat # 7428
Modified Oxford agar Base supplement	Neogen Corporation	Cat # 7991
Buffered *Listeria* enrichment broth	Neogen Corporation	Cat # 7675
Buffered *Listeria* enrichment broth supplement	Neogen Corporation	Cat # 7980
Tryptic Soy Broth	Difco/BD	Cat # DF0370-07-5
Brain heart infusion agar	Neogen Corporation	Cat # 7116
Pepsin	Sigma-Aldrich	Cat # 1.07185
Lipase	Sigma-Aldrich	Cat # L0382
Bovine Bile	Sigma-Aldrich	Cat # B8381
Porcine pancreatin	Sigma-Aldrich	Cat # P3292
DAPI	Cell Signaling	Cat # 4083
FITC-labeled 4 kDa dextran	Sigma-Aldrich	Cat # 46944
Anti-Listerial magnetic Dynabeads	Thermo Fischer Scientific	Cat # 71006

Halt proteases and phosphatase inhibitors	Thermo Fischer Scientific	Cat # PI78443
Restore Western Blot Stripping Buffer	Thermo Fischer Scientific	Cat # PI46430
Proteinase K	Dako	Cat # S3004
Bloxall solution (Blocking Solution)	Vector Labs	Cat # SP-6000
ImmPACT DAB (Chromogen)	Vector Labs	Cat # SK-4105
ImmPACT Vector Red (Chromogen)	Vector Labs	Cat # SK-5105
Prolong Gold (Antifade Reagent)	Invitrogen	Cat # P36934
**Critical Commercial Assays**		
Mouse TNF-α ELISA kit	Ray Biotech	Cat # ELM-TNFa-1
Mouse IL-6 ELISA kit	Ray Biotech	Cat # ELM-IL6-1
Mouse IFNγ ELISA kit	Ray Biotech	Cat # ELM-IFNg-1
BCA assay kit	Thermo Fisher Scientific	Cat # PI23235
B-PER extraction kit	Thermo Fisher Scientific	Cat # 78243
Mem-Per Plus Membrane Protein Extraction Kit	Thermo Fisher Scientific	Cat # PI89842
**Experimental Models: Cell Lines**		
Cell line: Caco-2	ATCC	Cat # HTB37
Cell line: Caco-2 presenting stable suppression of *hsp60* mRNA via short hairpin RNA (shRNA) mediated targeting	Our Lab[2]	N/A
Cell line: Caco-2 presenting a non-targeting control shRNA vector	Our Lab[2]	N/A
Cell line: Caco-2 presenting a constitutive overexpression of Hsp60	Our Lab[2]	N/A
Cell line: MDCK	ATCC	Cat # MDCK (NBL-2) CCL-34
**Experimental Models: Organisms/Strains**		
Mouse: A/J	The Jackson Laboratory	Stock # 000646
**Oligonucleotides**		
Lcas467 DNA Oligo, Alexa 488 5' /5Alex488N/CCGTCACGCCGACAACAG-3'	Integrated DNA Technologies[7]	N/A
LAPN-F 5'-GACCATGGATGGCAATTAAAGAAAATG-3' and LAPX-R-5'-GACTCGAGTCAAACACCTTTGTAAG-3'	Integrated DNA Technologies[6]	
**Software and Algorithms**		
Bio-Rad quantity one 4.6.9	Bio-Rad	http://www.bio-rad.com/en-ch/product/quantity-one-1-d-analysis-software
ImageJ 1.51t	NIH	https://imagej.nih.gov/ij/
GraphPad Prism 6.0	GraphPad Software	https://www.graphpad.com/scientific-software/prism/

Microsoft Excel 2010/365	Microsoft	https://products.office.com/en-us/excel
Nikon Elements Ver4.60.00	Nikon	https://www.nikoninstruments.com/Products/Software/NIS-Elements-Basic-Research

Supplementary References

1. Pandiripally, V.K., Westbrook, D.G., Sunki, G.R. & Bhunia, A.K. Surface protein p104 is involved in adhesion of *Listeria monocytogenes* to human intestinal cell line, Caco-2. *J. Med. Microbiol.* **48**, 117-124 (1999).
2. Burkholder, K.M. & Bhunia, A.K. *Listeria monocytogenes* uses *Listeria* adhesion protein (LAP) to promote bacterial transepithelial translocation, and induces expression of LAP receptor Hsp60. *Infect. Immun.* **78**, 5062-5073 (2010).
3. Bailey, T.W., do Nascimento, N.C. & Bhunia, A.K. Genome sequence of *Listeria monocytogenes* strain F4244, a 4b serotype. *Genome Announcements* **5**, e01324-01317 (2017).
4. Jagadeesan, B. et al. LAP, an alcohol acetaldehyde dehydrogenase enzyme in *Listeria* promotes bacterial adhesion to enterocyte-like Caco-2 cells only in pathogenic species. *Microbiology* **156**, 2782-2795 (2010).
5. Bhunia, A.K., Johnson, M.C. & Ray, B. Purification, characterization and antimicrobial spectrum of a bacteriocin produced by *Pediococcus acidilactici*. *J. Appl. Bacteriol.* **65**, 261-268 (1988).
6. Koo, O.K., Amalaradjou, M.A.R. & Bhunia, A.K. Recombinant probiotic expressing *Listeria* adhesion protein attenuates *Listeria monocytogenes* virulence in vitro. *PLoS One* **7**, e29277 (2012).
7. Quevedo, B. et al. Phylogenetic group-and species-specific oligonucleotide probes for single-cell detection of lactic acid bacteria in oral biofilms. *BMC Microbiol.* **11**, 14 (2011).
