# Supplementary material for: Receptor-targeted engineered probiotics mitigate lethal Listeria infection
Source: Nat Commun. 2020 Dec 11;11:6344. doi: 10.1038/s41467-020-20200-5 (PMC7732855; doi:10.1038/s41467-020-20200-5)
Supplement: Supplementary file 3 — Description of Additional Supplementary Files [file 41467_2020_20200_MOESM3_ESM.docx]

**Supplementary Video Legends**

**Supplementary Video 1.** The movie depicting the health status of naïve mice (mock-treated) without any probiotic or *Lm* challenge

**Supplementary Video 2.** The movie depicting the health status of naïve mice infected with *Lm* (48 hpi)

**Supplementary Video 3.** The movie depicting the health status of LbcWT-treated mice for 10 days followed by *Lm* (48 hpi)

**Supplementary Video 4****.** The movie depicting the health status of LbcLAP^Lm^-treated mice for 10 days followed by *Lm* challenge (48 hpi)

**Supplementary Video 5.** The movie depicting the health status of LbcLAP^Lin^-treated mice for 10 days followed by *Lm* challenge (48 hpi)
