# Supplementary material for: Receptor-targeted engineered probiotics mitigate lethal Listeria infection
Source: Nat Commun. 2020 Dec 11;11:6344. doi: 10.1038/s41467-020-20200-5 (PMC7732855; doi:10.1038/s41467-020-20200-5)
Supplement: Supplementary file 9 — Reporting Summary [file 41467_2020_20200_MOESM9_ESM.pdf]

nature research

Corresponding author(s): Professor Arun K. Bhunia

Last updated by author(s): Nov 17, 2020

Reporting Summary

Nature Research wishes to improve the reproducibility of the work that we publish. This form provides structure for consistency and transparency in reporting. For further information on Nature Research policies, see Authors & Referees and the Editorial Policy Checklist.

Statistics

For all statistical analyses, confirm that the following items are present in the figure legend, table legend, main text, or Methods section.

n/a	Confirmed	
☐	☒	The exact sample size (*n*) for each experimental group/condition, given as a discrete number and unit of measurement
☐	☒	A statement on whether measurements were taken from distinct samples or whether the same sample was measured repeatedly
☐	☒	The statistical test(s) used AND whether they are one- or two-sided<br>*Only common tests should be described solely by name; describe more complex techniques in the Methods section.*
☒	☐	A description of all covariates tested
☐	☒	A description of any assumptions or corrections, such as tests of normality and adjustment for multiple comparisons
☐	☒	A full description of the statistical parameters including central tendency (e.g. means) or other basic estimates (e.g. regression coefficient) AND variation (e.g. standard deviation) or associated estimates of uncertainty (e.g. confidence intervals)
☐	☒	For null hypothesis testing, the test statistic (e.g. *F*, *t*, *r*) with confidence intervals, effect sizes, degrees of freedom and *P* value noted<br>*Give P values as exact values whenever suitable.*
☒	☐	For Bayesian analysis, information on the choice of priors and Markov chain Monte Carlo settings
☒	☐	For hierarchical and complex designs, identification of the appropriate level for tests and full reporting of outcomes
☒	☐	Estimates of effect sizes (e.g. Cohen's *d*, Pearson's *r*), indicating how they were calculated

Our web collection on statistics for biologists contains articles on many of the points above.

Software and code

Policy information about availability of computer code

Data collection	Nikon Elements was used to collect immunofluorescence data. Leica Application Suite version X software was used to collect brightfield images.
Data analysis	Microscoft Excel 2010/365, ImageJ 1.51t, Graphpad Prism 6.0, Nikon AIR Elements Ver4.60.00, Bio Rad Quantity One 4.6.9, Leica Application Suite version X.

For manuscripts utilizing custom algorithms or software that are central to the research but not yet described in published literature, software must be made available to editors/reviewers. We strongly encourage code deposition in a community repository (e.g. GitHub). See the Nature Research guidelines for submitting code & software for further information.

Data

Policy information about availability of data

All manuscripts must include a data availability statement. This statement should provide the following information, where applicable:

- Accession codes, unique identifiers, or web links for publicly available datasets
- A list of figures that have associated raw data
- A description of any restrictions on data availability

The source data of all figures are provided in a “Source Data” file. All relevant data are available from the corresponding author upon request.

Field-specific reporting

Please select the one below that is the best fit for your research. If you are not sure, read the appropriate sections before making your selection.

☒ Life sciences ☐ Behavioural & social sciences ☐ Ecological, evolutionary & environmental sciences

Life sciences study design

All studies must disclose on these points even when the disclosure is negative.

Sample size	Sample sizes were based on previous experience with these types of experiments (Drolia et al. Cell Host Microbe, 2018, Burkholder & Bhunia. Infection and Immunity, 2010) . The analysis after each experiment verifies that the samples sizes were sufficient
Data exclusions	No data were excluded.
Replication	All data except figure 3 (1 replicate with n= 4-5 mice / time point and treatment) are representative of two or more independent experiments. Specifics are provided in each figure legend. All attempts at replication were successful.
Randomization	Randomization was used for mouse infections by using mice that were chosen randomly. For in vitro studies, randomized cell culture wells or biological samples were used.
Blinding	Investigators were blinded to group allocation during data collection.

Reporting for specific materials, systems and methods

We require information from authors about some types of materials, experimental systems and methods used in many studies. Here, indicate whether each material, system or method listed is relevant to your study. If you are not sure if a list item applies to your research, read the appropriate section before selecting a response.

Materials & experimental systems

n/a	Involved in the study	
☐	☒	Antibodies
☐	☒	Eukaryotic cell lines
☒	☐	Palaeontology
☐	☒	Animals and other organisms
☒	☐	Human research participants
☒	☐	Clinical data

Methods

n/a	Involved in the study	
☒	☐	ChIP-seq
☒	☐	Flow cytometry
☒	☐	MRI-based neuroimaging

Antibodies

Antibodies used

1. Mouse monoclonal anti-LAP; Our Lab; N/A
2. Rat monoclonal anti- ZO-1 Thermo Fisher Scientific Cat # MABT11MI, RRID: AB_628459
3. Rabbit polyclonal anti-Listeria Our Lab N/A
4. Rabbit polyclonal anti-Hsp60 Our Lab N/A
5. Mouse monoclonal anti-Hsp60 Enzo life Science Cat # ADI-SPA-806F, RRID: AB_11177888
6. Rabbit polyclonal anti- Muc-2 Novus Biological Cat # NBP1-31231, RRID: AB_10003763
7. Rabbit monoclonal anti- Ki67 Cell Marque Cat # 275R-15, RRID: AB_1158033
8. Rabbit polyclonal anti-Cleaved-caspase 3 (Asp175) Cell Signaling Cat # 9661S, RRID: AB_2341188
9. Mouse monoclonal anti-MLCK Sigma-Aldrich Cat # M7905, RRID: AB_477243
10. Rabbit polyclonal anti–phosphorylated MLC Cell Signaling Cat # 3671, RRID: AB_330248
11. Mouse monoclonal anti–phosphorylated MLC Cell Signaling Cat # 3675, RRID: AB_2250969
12. Mouse monoclonal anti-Claudin-1 Thermo Fisher Scientific Cat # 37-490-0, RRID: AB_ 2533323
13. Mouse monoclonal anti-Occludin Thermo Fisher Scientific Cat # 33-150-0, RRID: AB_2533101
14. Rabbit monoclonal anti-E-cadherin (Human) Cell Signaling Cat # 3195, RRID: AB_2291471
15. Rat monoclonal anti-E-cadherin (Mouse) Invitrogen Cat # 13-190-0, RRID: AB_2533005
16. Rabbit monoclonal anti-NF-κB p65 Cell Signaling Cat # 8242, RRID: AB_10859369
17. Rabbit monoclonal anti-Phospho-NF-κB p65 (Ser536) Cell Signaling Cat # 3033, RRID: AB_331284
18. Rat monoclonal anti-IL-10 Abcam Cat # AB 189392, RRID: AB_733113
19. Rat monoclonal anti-TGF-β clone (EPR21143) Abcam Cat # AB215715, RRID: NA
20. Rat monoclonal anti-F4/80 Bio-Rad Cat # MCA497R, RRID: AB_323279
21. Rat monoclonal anti-CD4 Invitrogen Cat # 14-9766-82, RRID: AB_2573008
22. Rabbit polyclonal anti-CD3 Dako Cat # A0452, RRID: AB_ 2335677
23. Rat monoclonal anti-CD8α Invitrogen Cat # 14-0808-82, RRID: AB_2572861
24. Rat monoclonal anti-FoxP3 Invitrogen Cat # 14-5773-82, RRID: AB_467576
25. Rabbit polyclonal anti-CD11c Invitrogen Cat # PA5-79537, RRID: AB_ 2746652
26. Goat polyclonal anti-NKp46 R&D Systems Cat # AF2225, RRID: AB_ 355192
27. Rabbit polyclonal anti-InlA Our Lab
28. Mouse monoclonal anti-N-acetylmuramidase (NamA) Our Lab
29. Mouse monoclonal anti-β-actin Santa Cruz Biotechnology Cat # sc-47778, RRID: AB_626632
30. Rabbit monoclonal anti-MEK 1/2 Cell Signaling Cat # 8727, RRID:AB_10829473
31. Normal rabbit IgG Santa Cruz Biotechnology Cat # sc-2027, RRID: AB_737197

32. Normal mouse IgG, Santa Cruz Biotechnology, Santa Cruz Biotechnology
33. Horse anti-mouse IgG (HRP-linked) Cell Signaling,Cat # 7076, RRID: AB_330924
34. Goat anti-rabbit IgG (HRP-linked)Cell Signaling Cat # 7074, RRID: AB_2099233
35. Goat anti-mouse IgG (H+L), F(ab')2 Fragment (Alexa Fluor 488 Conjugate) antibody, Cell Signaling,Cat # 4408, RRID: AB_10694704
36. Goat anti-rabbit IgG (H+L), F(ab')2 Fragment (Alexa Fluor 488 Conjugate) antibody Cell Signaling Cat # 4412, RRID: AB_1904025
37. Goat anti-mouse IgG (H+L), F(ab')2 Fragment (Alexa Fluor 555 Conjugate) antibody Cell Signaling Cat # 4409, RRID: AB_1904022
38.Goat anti-rabbit IgG (H+L), F(ab')2 Fragment (Alexa Fluor 555 Conjugate) antibody Cell Signaling Cat # 4413, RRID: AB_10694110
39. Goat anti-rat IgG (H+L), (Alexa Fluor 555 Conjugate) antibody Cell Signaling Cat # 4417, RRID: AB_10696896
40. Goat anti-rabbit IgG (H+L), Highly Cross-Adsorbed (Alexa Fluor 647 Conjugate) antibody Invitrogen Cat # A21245, RRID: AB_253813
41. Goat anti-rabbit ImmPRESS HRP Vector Labs Cat # MP-7451, RRID: AB_ 2631198
42. Goat anti-rat ImmPRESS HRP (M adsorbed) Vector Labs Cat # MP-7444, RRID: AB_ 2336530
43. Horse anti-mouse ImmPRESS HRP Vector Labs Cat # MPX-2402, RRID: AB_ 2336831
44. Horse anti-Goat ImmPRESS HRP Vector Labs Cat # MP-7405, RRID: AB_ 2336526
45. Isotype control Mouse IgG Vector Labs Cat # I-2000, RRID: AB_ 2336354
46. Isotype control Rabbit IgG Vector Labs Cat # I-1000, RRID: AB_ 2336355
47. Isotype control Rat IgG Vector Labs Cat # I-4000, RRID: AB_ 2336356

Validation

Mouse monoclonal anti-LAP and Rabbit polyclonal anti-InlA: Has been validated in our lab by western blot against recombinant LAP and InlA, respectively and listeria whole-cell lysates and showing that it only reacts with LAP and InlA, respectively (Drolia et al. Cell Host Microbe, 2018) (Burkholder & Bhunia. Infection and Immunity, 2010)
Rat monoclonal anti- ZO-1: is described by the manufacturer to be quality control tested using immunofluorescent staining and western blotting. Also has been validated in our lab by immunofluorescence staining (Drolia et al. Cell Host Microbe, 2018)
Rabbit polyclonal anti-Listeria: Has been validated in our lab by western blot against listeria whole-cell lysates and immunofluorescence. (Drolia et al. Cell Host Microbe, 2018)
Rabbit polyclonal anti-Hsp60: Has been validated in our lab by western blot against Caco-2 whole-cell lysates and immunofluorescence. (Burkholder & Bhunia. Infection and Immunity, 2010)
Mouse monoclonal anti-Hsp60: is described by the manufacturer to be quality control tested using immunohistochemistry staining and western blotting. Has also been validated in our lab by western blot and immunofluorescence against Hsp60 KD and over-expression cell lines. (Drolia et al. Cell Host Microbe, 2018) and (Burkholder & Bhunia. Infection and Immunity, 2010)
Mouse monoclonal anti-MLCK: Has been validated in detail using western blot and by functional depletion of myosin light chain kinase activity from intestinal epithelial cell lysates (Clayburgh et al. J Biol Chem, 2005).
Rabbit polyclonal anti–phosphorylated MLC Cell Signaling: is described by the manufacturer to be quality control tested using immunofluorescence staining and western blotting. Has also been validated in our lab by western blot and immunofluorescence staining (Drolia et al. Cell Host Microbe, 2018)
Mouse monoclonal anti–phosphorylated MLC: is described by the manufacturer to be quality control tested using immunofluorescence staining and western blotting and for reactivity in human and mouse species.
Mouse monoclonal anti-Claudin-1: is described by the manufacturer to be quality control tested using immunofluorescence staining and western blotting for reactivity in human and mouse species.
Mouse monoclonal anti-Occludin: is s described by the manufacturer to be quality control tested using immunofluorescence staining and western blotting. Occludin antibodies were also been previously validated using occludin KD cell lines. Buschmann, Mary M., et al. Molecular biology of the cell, 2013.
Rabbit monoclonal anti-E-cadherin (Human) Cell Signaling: is described by the manufacturer to be quality control tested using immunofluorescence staining and western blotting. Has also been validated in our lab by western blot and immunofluorescence staining (Drolia et al. Cell Host Microbe, 2018)
Rat monoclonal anti-E-cadherin (Mouse): is described by the manufacturer to be quality control tested using immunofluorescence staining and western blotting. Has also been validated in our lab by western blot and immunofluorescence staining.
Rabbit monoclonal anti-NF-κB p65: is described by the manufacturer to be quality control tested using immunofluorescence staining and western blotting. Has also been validated in our lab by western blot and immunofluorescence staining (Drolia et al. Cell Host Microbe, 2018)
Rabbit monoclonal anti-Phospho-NF-κB p65 (Ser536): is described by the manufacturer to be quality control tested using immunofluorescence staining and western blotting. Has also been validated in our lab by western blot and immunofluorescence staining (Drolia et al. Cell Host Microbe, 2018)
All other antibodies have been described by the manufacturer to be quality control tested using immunohistochemistry or immunofluorescence staining or western blotting. We have validated all these antibodies by using tissue-arrays immunohistochemistry and respective isotype controls. All secondary antibodies have been described by the manufacturer to be quality control tested for species specific use.

Eukaryotic cell lines

Policy information about cell lines

Cell line source(s)

The human colon carcinoma Caco-2 cell line (ATCC # HTB37) from 25-35 passages were used. The Caco-2 cells presenting stable suppression of hsp60 mRNA (Hsp60–), or presenting a non-targeting control shRNA vector (Hsp60Vec) or exhibiting a constitutive over-expression of Hsp60 were previously developed (Hsp60+) in our lab (Burkholder and Bhunia Infection &

	Immunity 2010.<br>The MDCK cell line (ATCC # NBL-2 CCL-34) from 10-20 passages were used.
Authentication	The cell line was authenticated on the basis of morphology, basal barrier function, and loss of barrier function by cytokines.
Mycoplasma contamination	Cells were routinely tested for mycoplasma contamination and were negative throughout the course of these studies.
Commonly misidentified lines<br>(See ICLAC register)	None Used

Animals and other organisms

Policy information about studies involving animals; ARRIVE guidelines recommended for reporting animal research

Laboratory animals	Female mice (Strain: A/J, 6-8 weeks of age, Stock # 000645 purchased from the Jackson Laboratories)
Wild animals	None Used
Field-collected samples	None Used
Ethics oversight	The animal procedure was approved by the Purdue University Animal Care and Use Committee (PACAUC approval No.1201000595) who adheres to the recommendations of the Guide for the Care and Use of Laboratory Animals published by the National Institute of Health.

Note that full information on the approval of the study protocol must also be provided in the manuscript.
